# Spatial Disparity in Food Environment and Household Economic Resources Related to Food Insecurity in Rural Korean Households with Older Adults

**DOI:** 10.3390/nu10101514

**Published:** 2018-10-16

**Authors:** Jae Eun Shim, Seo-jin Kim, Kirang Kim, Ji-Yun Hwang

**Affiliations:** 1 Department of Food and Nutrition, Daejeon University, Daejeon 34520, Korea; jshim@dju.kr; 2 Department of Foodservice Management and Nutrition, Sangmyung University, Seoul 03016, Korea; kseojin2@gmail.com; 3 Department of Food Science and Nutrition, Dankook University, Cheonan 31116, Korea

**Keywords:** food insecurity, food environment, spatial disparity, older adults, rural area

## Abstract

Different contextual factors of a household and a community, such as access to resources and transportation, may influence the level of food insecurity. The objective of this study was to identify how food environmental factors and economic resources were related to food insecurity in Korean older adults residing in different contexts of rural areas. Face-to-face interviews with 248 older adults residing in land (*n* = 149) and mountain (*n* = 99) rural areas were performed. In both areas, risk of food insecurity was increased for households with limited community food accessibility measured by having difficulties in food purchasing due to food stores far from home. There were discrepancies in factors related to increased risks of food insecurity between households in land and mountain areas. The experience of reducing food expenditure resulting from burden of heating costs during the winter in households in a mountain area whereas the percent proportion of housing fee and household cook’s physical disability in households residing in the land area were found to be factors associated with increased risks of food insecurity. For households residing in mountain areas, the risk of food insecurity was decreased when economic resources measured by average monthly income for the last one year was increased and there was farming or home gardening activity. Such spatial disparity might affect household food insecurity in rural areas. In addition, food environmental factors and economic resources may affect household food insecurity differently according to the diverse contexts of rural areas. Better understanding of spatial challenges in food insecurity faced by seniors in a large rural area would help prepare programs or policy change to strengthen and improve their food environments.

## 1. Introduction

Food environment has been a significant determinant of nutrition and health status [1,2,3,4]. Among food environment factors, poor access to food due to limited economic resources, transportation difficulty, and distance to a food grocery are important factors affecting food security and food consumption among older adults with mobility limitations [5,6,7,8]. Problems resulting from limited food access are more common in rural seniors than those in urban seniors [6,9,10]. This spatial disparity could be partially explained by differential access to community food environments, such as a greater distance from home to food stores and lack of transportation related to access in rural areas [9,11,12]. However, little work has been done on the spatial disparity in food insecurity for older adults who reside in different contexts of rural areas.

The nationwide prevalence of food insecurity in Korea in 2012 was 11.3%, with 13.3% in older adults [13]. Especially, the nationwide prevalence of food insecurity in low-income households with elders aged ≥65 years was 56.2% [14]. Korean older adults are more likely to be food-insecure if they are living alone, receiving national basic livelihood security benefits, have poor subjective health status, have low levels of private transfer income, do not consume self-produced food, and do not use free meal services [15,16]. The proportion of food insecurity among Korean older adults has been found to be higher in urban areas than that in rural areas [13,16], showing opposite results from other countries [6,12,17]. Different results between areas could be explained by geographic isolation, community resources, and household economic resources related to food expenditures, including housing status, heating and medical expenditures, and social support [5,18,19,20].

Although the attention toward food environments is increasing, little is known about which factors of food environment and economic resources might serve as barriers or facilitators for food security among rural seniors who do not have the same personal resources or access to food environments. In addition, there is little study that simultaneously examines two factors to explain their food insecurity, especially in an Asian context. Better understanding of spatial challenges in food insecurity faced by seniors in a large rural area would help prepare programs or policy change to strengthen and improve food environments. Therefore, the objective of this study was to identify how food environmental factors and economic resources were related to food insecurity in Korean older adults residing in different contexts of rural areas.

## 2. Methods and Materials

### 2.1. Study Design and Subjects

The study population was selected from Yangpyeong County in Gyeonggi Province and Hongcheon County in Gangwon Province. Two targeted areas were determined by the review of experts in geography, nutrition, and public health to represent the different characteristics of rural areas, such as land and mountain areas. Yangpyeng County is located 45 km east of Seoul, the capital of South Korea. It is a designated farming area with regulatory exemptions pursuant to the Environment-Friendly Agriculture Fosterage Act. Hongcheon County is located 81 km northeast of Seoul. It is a mountainous region branching out from the Baegdu Mountain range located at the center of the Korean Peninsula that forms a canyon. In this study, Yangpyeong County is referred to as a land area while Hongcheon County is referred to as a mountain area.

The study sample was recruited by National Home Healthcare Services (NHHS) nurses. The NHHS has been implemented to promote health for vulnerable populations with health problems by home visiting nurses in the public center [21]. Registration for NHHS is limited by the criteria of family income levels [21]. The recruitment strategy consisted of two steps. First, home visiting nurses recruited older adults aged 65 years or more without a disability or cognitive impairment. Second, the contact information of eligible households was transferred to the survey team composed of professionally experienced interviewers if subjects agreed to participate in the survey. Two heads of interviewers were trained with a standardized study protocol for this study. They trained all interviewers for two days before the survey. A total of 248 households (149 from the land area and 99 from the mountain area) completed this survey. Informed consent was obtained voluntarily from all subjects. This study was approved by the Institutional Review Board of Sangmyung University, Seoul, Korea (BE2013-8).

### 2.2. Methods and Variables

The survey was conducted with a household member in charge of cooking. A structured questionnaire, including information on general characteristics, household economic factors, food environment factors, and food insecurity, was administered by trained interviewers. General characteristics included demographic factors, such as age, sex, education status, job status, family type, health status, and beneficiary of national basic livelihood. Economic and food environment factors were examined by objective and perceived (subjective) measures.

#### 2.2.1. Household Economic Factors

Objective economic factors included monthly incomes and expenditures from food, housing, heating, and medical presented in US dollars using the exchange rate of currency (1125 Korean won = 1 U.S. dollar). The proportion of expenditure from each item out of the total expenditure was also calculated. For the perceived index of economic factors, subjects were asked if they had an experience of reducing food expenditure due to the burden of housing fees or heating costs during a winter season.

#### 2.2.2. Food Environment Factors

Food environment conceptualizations in this study have been developed to specifically reflect a conceptual model of food availability, access, and utilization in the context of food security [22]. Several previous studies have explored the best measurement concerning the definition of a food environment [23,24,25]. Generally, key concepts of a food environment have been considered as food availability and accessibility, which refer to the adequacy of the supply of healthy food and geographic food access, respectively [23,24,25]. A variety of measurement tools of food environments has been also developed to describe characteristics of environments, including objective and respondent-based perceived measures [9,24,25]. Specifically, the community and household level food environment factors, which are the focus of this study, were based on the conceptual model of food environment developed by Glanz and colleagues [23]. The model organizes food environment features into the community food environment (the distribution of food sources within a community), the organizational food environment (the multiple settings where people eat or procure food), and the consumer food environment (available healthy foods and the cost and quality of foods in local food outlets). Therefore, this study examined the availability and accessibility of community and household level environments using both objective and perceived measures to assess the food environment features.

Household food availability was assessed by objective measures asking ways of food acquisition: (1) Purchasing food, (2) farming or home gardening, (3) tangible private supports related to food acquisition, and (4) participation in public food assistance programs. Household food accessibility was assessed by three questions: (1) Whether a family member in charging of cooking had a physical disability, (2) whether subjects obtained intangible support related to food purchasing from family, and (3) whether subjects had intangible support related to food purchasing from neighbors.

Community food availability and accessibility were assessed by both objective and perceived measures. Community food availability was examined by asking places where subjects mostly purchased foods and their perception on whether the nearest food store had a variety of foods that could be purchased. For community food accessibility, transportation and minutes to the nearest food stores were examined as objective measures. Perceived community food accessibility was assessed using three items to determine whether subjects perceived difficulty in food purchasing due to the following reasons: (1) Long distance of food stores from home, (2) bus stop far from home, or (3) inconvenient bus route.

#### 2.2.3. Household Food Insecurity

Korean Household Food Security Survey Module (K-HFSS) from the Korea National Health Examination and Nutrition Survey, which has been validated in previous studies [26,27], was used to determine food insecurity. The K-HFSS was based on the eighteen-item US Household Food Security Survey Module (HFSSM). The 18-item questionnaire was composed of 3 household-referenced questions, 7 adult-referenced questions, and 8 child-referenced questions. In this study, the adult food security survey module, which consists of a 10-item questionnaire without 8 child-referenced questions, was completed by each household. A score of 1 indicating food insecure conditions was assigned to affirmative responses while a score of 0 was assigned to all other responses in each questionnaire. Subjects were classified into a food security group if they had scores of 0–2 and a food insecurity group if they had scores of 3–10 by the classification criteria of the adult food security survey module [26].

### 2.3. Data Analysis

Data are presented as percentage and a number for categorical variables and mean ± standard deviation (SD) for continuous variables. Results between rural areas were compared using a Chi-square test for categorical variables and a *t*-test for continuous variables. Odds ratios and 95% confidence intervals for food insecurity were calculated using a logistic regression model to examine economic and food environmental characteristics affecting food insecurity in each area. Stepwise logistic regression was used to identify the most explainable economic and food environmental factors. Potential independent variables of the multivariate model were selected based on a significant relationship with food insecurity in each area. All analyses were performed using IBM SPSS Statistics 23 (IBM Company, Armonk, NY, USA), and *p*-values < 0.05 were considered statistically significant.

## 3. Results

### 3.1. General Characteristics of Households

General characteristics of households between two areas are shown in Table 1. The prevalence of food insecurity was 40.4% in the mountain area and 46.3% in the land area, showing no significant difference between the two areas. The mean age of the family member in charge of cooking in the household was about 76 years old in the mountain area and 77 years old in the land area. Households in the mountain area had a lower percentage of single-person households (63.6% vs. 77.9%) and beneficiaries of the national basic livelihood (36.4% vs. 59.1%), with a higher percentage of household cooks with a low education level (97% vs. 79.2%) compared to those in the land area. In each study area, the percentage of households of a family size larger than two was less than 5%. All the household members were older adults in 47.4% of the two person households, and 5.2% of the two person households included adolescents.

### 3.2. Economic Factors Related to Food Insecurity

Economic factors related to food insecurity according to areas are shown in Table 2. For objective economic indices, average household monthly income was not significantly different between the two areas ($282.1 for the mountain area and $291.3 for the land area). However, average household monthly expenditure was higher in the land area than that in the mountain area ($178.4 vs. $146.9). Incomes from earnings and allowance from family were higher in households in the mountain area while incomes from subsidies were higher in households in the land area. Among expenditures, the expenditure from housing fees and heating costs was higher in the land area while the expenditure from medical expenses was higher in the mountain area. The proportion of expenditure from heating costs out of total expenditures was the highest in both areas (33.7% for the mountain area and 38.7% for the land area), followed by the proportion of food expenditure.

Perceived economic characteristics were significantly different between the two areas. The proportion of households with an experience of reducing food expenditure due to the burden of housing fees or heating costs during a winter season was higher in the land area than that in the mountain area. One-third of households in the land area experienced a reduction of food expenditure due to the burden of housing fees, and about 70% of households in the land area experienced a reduction of food expenditure due to heating costs.

In terms of factors affecting food insecurity in each area, households with an experience of reducing food expenditure due to the burden of heating costs during a winter season were more likely to have food insecurity than those without such an experience in mountain areas, showing an odds ratio (OR) of 6.48 (95% confidence intervals (CI) = 2.119–19.814). For land areas, average monthly expenditure for the last one year (OR = 1.005, 95% CI = 1.002–1.008), expenditure from housing fees (OR = 1.008, 95% CI = 1.003–1.012), and experience of reducing food expenditure due to the burden of housing fees (OR = 2.943, 95% CI = 1.463–5.919) or heating costs during a winter season (OR = 2.725, 95% CI = 1.284–5.784) showed significantly increased risk of food insecurity.

### 3.3. Food Environment Factors Related to Food Insecurity

Results of food environments at household and community levels related to food insecurity according to areas are shown in Table 3. Proportions of households that acquired foods by money and public food assistance programs were significantly higher in the land area than those in the mountain area (87.9% vs. 78.8% for food purchases and 68.5% vs. 39.4% for the public food assistance program). The proportion of households that acquired foods by farming or home gardening and tangible private support was higher in the mountain area than that in the land area. However, the difference between the two was not statistically significant. Household food accessibility factors of the household cook’s physical disability and intangible private support for food purchasing were not significantly different between the two areas.

For community food availability, both areas had the highest proportion of households using discount stores to buy foods (42.4% in the mountain area and 53.7% in the land area). The proportion of respondents using a supermarket was significantly higher in the mountain area. For community food accessibility, transportation concerns measured by objective measures between the two areas were not significantly different. Perceptions on long distances to food stores or limited access to transportation were not significantly different between the two areas either. However, the proportion of subjects who perceived difficulties in food purchasing was higher in the land area compared to that in the mountain area.

Perceptions on long distances to food stores was a significant risk factor of food environments for food insecurity in both areas (OR = 9.647, 95% CI = 2.811–33.101 for the mountain area; OR = 3.6, 95% CI = 1.686–7.684 for the land area). For the mountain area, purchasing foods in a traditional market was further related to a decreased risk of food insecurity (OR = 0.337, 95% CI = 0.121–0.934). For the land area, households with a disabled family member who was responsible for cooking and those with a perception on no variety in types of foods in the nearest food stores were more likely to have food insecurity compared to households without such factors (OR = 2.579, 95% CI = 1.066–6.240; and OR = 3.9, 95% CI = 1.186–12.821, respectively).

### 3.4. The Most Explainable Economic and Food Environmental Factors Related to Food Insecurity in Each Area

To identify the most explainable economic and food environmental factors related to food insecurity in each area, a stepwise logistic regression was conducted. As shown in Table 4, factors increasing the risk of food insecurity in the households residing in the mountain area were experiences of reducing food expenditure resulting from the burden of heating costs during the winter (OR = 5.664 95% CI = 1.518–21.136) and having difficulties in purchasing food due to food stores far from home (OR = 36.3, 95% CI = 3.99–330.48). Factors related to a decreased risk of their food insecurity were the average monthly income for the last one year (OR = 0.995, 95% CI = 0.991–0.999) and farming or home gardening (OR = 0.022, 95% CI = 0.002–0.283). In terms of households residing in the land area, significant factors related to food insecurity were the percent proportion of housing fees (OR = 1.032, 95% CI = 1.01–1.036), household cook’s physical disability (OR = 2.846, 95% CI = 1.074–7.538), and having difficulties in food purchasing due to food stores being far from home (OR = 4.675, 95% CI = 2.05–10.66). All factors contributing to food insecurity in the land area were related to an increased risk of food insecurity.

## 4. Discussion

We found a strong association between limited community food accessibility, measured by having difficulties in food purchasing due to food stores being far from home, and risk of food insecurity in both mountain and land rural areas. In addition, there were discrepancies in factors related to increased risks of food insecurity between the two areas: Subjective economic indices (the experience of reducing food expenditure resulting from the burden of heating costs during the winter in households) in mountain areas and household expenditure (the percent proportion of housing fees) and household food accessibility (household cook’s physical disability) in land areas. For households residing in mountain areas, the risk of food insecurity was decreased with economic resources (average monthly income for the last one year) and household food availability (farming or home gardening). Our results suggest that spatial disparity might affect household food insecurity in rural areas. However, differential effects may exist according to diverse contextual environments and economic resources. Our finding is consistent with previously reported results [7,9,10,11,26,28]. The food environment has key features that can affect the food choices of older adults. One such characteristic is food accessibility [11,12]. Seniors living in rural areas have lower access to healthy food than their urban or suburban counterparts due to mobility disabilities [7], low incomes without ownership of a vehicle [7], relatively high cost of healthy food items [28], and limited access to food stores [10,11] or food selection [28]. In contrast, certain features of food environments in rural areas provide protection against food insecurity, such as farming, home gardening, and other types of household food production [28]. These previous results are fairly consistent with results of the present study, showing that food insecurity was increased when there were difficulties in food purchase due to food stores being far from home in both land and mountain areas. Food insecurity was also increased when a household member in charge of cooking had a physical disability in land areas. However, it was decreased when seniors practiced farming and home gardening in mountain areas.

Economic expenditures were negatively related to food insecurity in our subjects, although there were inconsistencies between the two different areas: Food insecurity was increased when households experienced a reduction in food expenditure due to increased heating costs during the winter in mountain areas and when the percent proportion of housing fees to total expenditure was increased in land areas. Little is known about non-food based strategies that affect food security in rural seniors. Results from the present study suggest that several potential causes and solutions to food access for rural seniors should be considered within this context. Older adults were unable to accommodate food needed due to an expansion of expenditure from contextually distinct characteristics. Furthermore, household food availability or accessibility was not improved by private or public assistance, but by the own features of households, such as home food production. In this regard, a regionally tailored, longitudinal intervention study is needed due to the complex nature and long latency of any effect. In addition, our findings revealed the need of including spatial variables in future analyses of the causality of food insecurity among the underprivileged, such as older adults, and for identifying suitable policy reactions.

This study had several limitations that should be addressed in future studies. An association of food insecurity with food environments and economic resources was observed only in a cross-sectional setting. The causality between spatial disparity in food environments and household economic resources should be evaluated in a future study. Furthermore, the nonrandom sampling scheme and a limited sample size made it difficult to generalize results of this study to the larger population. Our results therefore need to be confirmed with larger and wider studies using more representative samples of seniors from diverse contextual settings. Nonetheless, our study had several strengths. To date, an association between discrete food environments, such as land and mountain areas, and food insecurity has been examined mostly in poor families with children in developing countries. Furthermore, the majority of putative risk factors of economic resources and food environments related to food insecurity were included in our analysis.

In conclusion, our results suggest that spatial disparity may affect household food insecurity in rural areas. In addition, food environmental factors and economic resources may affect household food insecurity differently according to diverse contextual rural areas. As next steps, it would be helpful to explore the design of contextually tailored interventions to eliminate or reduce barriers in the rural context to increase food access for older adults that experience limited income resources, but have a relatively high expenditure, physical impairment, and stores that do not accommodate their needs.

## Figures and Tables

**Table 1:** General characteristics of households between different rural areas.

Characteristics	Mountain Area		Land Area		*p*-Value
	*n* = 99		*n* = 149		
	%	*n*	%	*n*	
Food insecure households	40.4	40	46.3	69	0.3589
Family size					0.0349
1	63.6	63	77.9	116	
2	32.3	32	18.1	27	
≥3	4.0	4	4.0	6	
Beneficiaries of national basic livelihood	36.4	36	59.1	88	0.0001
Cook of the household					
Age (years) ^1^	75.7 ± 7.8		76.7 ± 7.3		0.3357
Women	86.9	86	79.2	118	0.1213
≤6 years of primary education	97.0	96	79.2	118	<0.0001

^1^ mean ± standard deviation (SD).

**Table 2:** Economic characteristics of households and risks for food insecurities according to different rural areas.

Characteristics	Mountain Area (*n* = 99)					Land Area (*n* = 149)				
	Values		Odds Ratios ^1^	95% Confidence Limits		Values		Odds Ratios ^1^	95% Confidence Limits	
*Objective economic indices*										
Average monthly income for last 1 year, $ ^2^	281.2 ± 158.9		0.998	0.995	1.001	291.3 ± 147.6		0.999	0.997	1.002
Earnings ^3^	55.1 ± 116.3		0.998	0.994	1.002	22.8 ± 73.5		0.996	0.990	1.002
Subsidies ^3^	172.0 ± 134.8		1.000	0.997	1.003	241.0 ± 151.2		1.001	0.999	1.003
Allowances from family ^3^	54.1 ± 91.9		1.000	0.997	1.003	27.5 ± 78.0		1.001	0.999	1.003
Average monthly expenditure for last 1 year, $ ^3^	146.9 ± 108.0		1.001	0.997	1.005	178.4 ± 109.6		1.005	1.002	1.008
Food expenses	42.2 ± 55.5		1.001	0.994	1.008	46.5 ± 47.8		1.001	0.994	1.007
Housing fee	39.4 ± 61.7		1.005	0.999	1.012	55.6 ± 82.1		1.008	1.003	1.012
Heating costs ^3^	35.1 ± 23.1		0.986	0.968	1.004	56.3 ± 40.2		1.005	0.996	1.013
Medical expense ^3^	30.1 ± 47.9					20.0 ± 32.4				
% Proportions of expenditure components, all year										
Food expenditure	27.3 ± 20.9		0.995	0.975	1.014	26.6 ± 21.5		0.986	0.971	1.002
Housing fee	20.0 ± 29.1		1.010	0.996	1.024	21.3 ± 29.3		1.021	1.009	1.033
Heating costs	33.7 ± 27.6		0.994	0.980	1.009	38.7 ± 25.0		0.987	0.974	1.001
Medical expenditure	18.0 ± 23.0		0.992	0.974	1.010	12.7 ± 19.7		0.996	0.980	1.013
*Subjective economic indices* ^4^										
Experience of reducing food expenditure resulting from burden of housing fee ^3^	5.1	(5)	-	-	-	34.9	(52)	2.943 ^5^	1.463	5.919
Experience of reducing food expenditure resulting from burden of heating costs during the winter ^3^	20.2	(20)	6.480 ^5^	2.119	19.814	70.5	(105)	2.725 ^5^	1.284	5.784

Mean ± SD. ^1^ Risks for food insecurities. ^2^ The exchange rate of currency was 1125 Korean won per 1 US dollar. ^3^ Significant difference between two areas by *t*-test or Chi-square test (*p* < 0.05). ^4^ Values were % (*n*). ^5^ Reference ‘no’.

**Table 3:** Food environmental characteristics of households and risks for food insecurities according to rural area.

Characteristics	Mountain Area (*n* = 99)						Land Area (*n* = 149)					
	Values			Odds Ratios ^1^	95% Confidence Limits		Values			Odds Ratios ^1^	95% Confidence Limits	
	%		*n*				%		*n*			
*Household food availability*												
Purchasing food ^2^	78.8		78	1.467	0.533	4.036	87.9		131	1.412	0.515	3.868
Farming or home gardening	24.2		24	0.301	0.102	0.890	18.1		27	0.421	0.171	1.035
Getting tangible private support related to food acquisition	22.2		22	1.027	0.392	2.694	14.8		22	0.960	0.387	2.383
Participating in public food assistance program ^2^	39.4		39	2.105	0.922	4.809	68.5		102	1.417	0.704	2.854
*Household food accessibility*												
Household cook’s physical disability	19.2		19	1.852	0.676	5.074	17.5		26	2.579	1.066	6.240
Getting intangible support from family for purchasing food	9.1		9	0.350	0.068	1.808	10.1		15	0.970	0.330	2.852
Getting intangible support from neighbors for purchasing food	9.1		9	3.111	0.717	13.501	8.1		12	0.777	0.233	2.586
*Community food availability, objective indices*												
Places to purchase food												
Traditional market	27.3		27	0.337	0.121	0.934	36.2		54	1.200	0.598	2.409
Supermarket ^2^	25.3		25	1.787	0.684	4.674	10.1		15	0.970	0.330	2.852
Super Supermarket	42.4		42	0.688	0.279	1.697	53.7		80	0.787	0.389	1.590
*Community food availability, perception*												
No various foods in the nearest food store	7.1		7	1.025	0.213	4.923	10.7		16	3.900	1.186	12.821
*Community food accessibility, objective indices*												
Transportation to the nearest food stores												
By walk	45.5		45	1.000			46.3		69	1.000		
By driving	33.3		33	0.653	0.260	1.638	41.6		62	0.749	0.376	1.493
Not applicable	21.2		21	0.571	0.194	1.683	12.1		18	0.618	0.214	1.782
Distance to the nearest food stores (min) ^3^												
By walk	15.0	±	9.7	1.025	0.963	1.090	19.6	±	11.7	1.017	0.975	1.06
By driving	22.4	±	11.5	1.021	0.960	1.086	16.3	±	7.8	0.955	0.891	1.023
*Community food accessibility, perception*												
Having difficulties in food purchasing due to food stores far from home	20.2		20	9.647	2.811	33.101	30.9		46	3.600	1.686	7.684
Having difficulties in food purchasing due to bus stop far from home	15.2		15	0.889	0.282	2.799	18.1		27	0.864	0.369	2.023
Having difficulties in food purchasing due to inconvenience of bus route	5.1		5	0.903	0.142	5.735	5.4		8	0.145	0.017	1.216

^1^ Risks for food insecurities, reference ‘no’ except ‘distance to the nearest food stores’ and ‘transportation to the nearest food stores’. ^2^ Significant difference between two areas by Chi-square test (*p* < 0.05). ^3^ Mean ± SD.

**Table 4:** Risk factors for food insecurities in two rural areas with different demographic, economic, and food environment factors.

Risk Factors	Odds Ratios	95% Confidence Limits	
*Mountain area*			
*Objective economic indices*			
Average monthly income for last 1 year, $	0.995	0.991	0.999
*Subjective economic indices*			
Experience of reducing food expenditure resulting from burden of heating costs during the winter (reference ‘no’)	5.664	1.518	21.136
*Household food availability*			
Farming or home gardening (reference ‘no’)	0.022	0.002	0.283
*Community food accessibility, perception*			
Having difficulties in food purchasing due to food stores far from home (reference ‘no’)	48.58	4.83	488.69
*Land Area*			
*Objective economic indices*			
Percent proportion of housing fee	1.023	1.01	1.036
*Household food accessibility*			
Household cook’s physical disability (reference ‘no’)	2.846	1.074	7.538
*Community food accessibility, perception*			
Having difficulties in food purchasing due to food stores far from home (reference ‘no’)	4.675	2.05	10.66

Odds ratios and 95% confidence limits of risk factors for households’ food insecurity. A stepwise approach was applied to select the most explainable risk factors in a model with potential independent variables that were selected based on their association with food insecurities in each area (α = 0.15). Potential independent variables in multivariate models for the mountain area were the education years of the households’ cook (<6 years or ≥6 years), average monthly income, experience of reducing food expenditure resulting from the burden of non-food expenses, such as housing fees or heating costs in winter, food acquisition by farming/ home gardening (yes or no), participating in a public food assistance program (yes or no), purchasing foods in a transitional market (yes or no), or difficulties in food purchasing due to food stores being far from home (yes or no); potential independent variables in multivariate models for the land area were the education years of the households’ cook (<6 years or ≥6 years), households’ income with earnings ($), average monthly expenditure ($), percent proportions of housing fees, percent proportions of heating costs, percent proportions of medical expenditures, experience of reducing food expenditure resulting from the burden of non-food expenses, such as housing fees (yes or no) or heating costs in winter (yes or no), food acquisition by farming/ home gardening (yes or no), physical disabilities of the households’ cook, no variety of foods in the nearest food store, difficulties in food purchasing due to food stores being far from home (yes or no), or inconvenient bus route (yes or no).
